# Inter-center comparison of EasyTube and endotracheal tube during general anesthesia in minor elective surgery

**DOI:** 10.1371/journal.pone.0178756

**Published:** 2017-06-02

**Authors:** Oliver Robak, Sonia Vaida, Mostafa Somri, Luis Gaitini, Lisa Füreder, Michael Frass, Lukasz Szarpak

**Affiliations:** 1 Department of Medicine I, Medical University of Vienna, Vienna, Austria; 2 Department of Anesthesiology, Penn State Milton S. Hershey Medical Centre, Hershey, PA, United States of America; 3 Department of Anesthesiology, Bnai Zion Medical Centre, Haifa, Israel; 4 Division of Cardiothoracic and Vascular Anesthesia and Intensive Care Medicine, Department of Anesthesia, General Intensive Care and Pain Medicine, Medical University of Vienna, Vienna, Austria; 5 Department of Emergency Medicine, Medical University of Warsaw, Warsaw, Poland; Scuola Superiore Sant'Anna, ITALY

## Abstract

**Background:**

The EasyTube^®^ (EzT) is a supraglottic airway device (SAD) enabling ventilation irrespective of its placement into the esophagus or trachea. Data obtained on SADs from multicenter studies, performed in highly specialized centers cannot always be transferred to other sites. However, data on comparability of different sites are scarce. This study focused on inter-site variability of ventilatory and safety parameters during general anesthesia with the EzT.

**Methods:**

400 patients with ASA physical status I-II undergoing general anesthesia for elective surgery in four medical centers (EzT group (n = 200), ETT group (n = 200)). Mallampati classification, success of insertion, insertion time, duration of ventilation, number of insertion attempts, ease of insertion, tidal volumes, leakage, hemodynamic parameters, oxygenation, and complications rates with the EasyTube (EzT) or endotracheal tube (ETT) in comparison within the sites and in between the sites were recorded.

**Results:**

Intra-site and inter-site comparison of insertion success as primary outcome did not differ significantly. The inter-site comparison of expiratory minute volumes showed that the volumes achieved over the course of anesthesia did not differ significantly, however, mean leakage at one site was significantly higher with the EzT (0.63 l/min, p = 0.02). No significant inter-site differences in heart rate, blood pressure, or oxygenation were observed. Sore throat and blood on the cuff after removal of the device were the most frequent complications with significantly more complications at one site with the EzT (p = 0.01) where insertion was also reported significantly more difficult (p = 0.02).

**Conclusion:**

Performance of the EzT but not the ETT varied between sites with regard to insertion difficulty, leakage, and complications but not insertion success, ventilation, hemodynamics, and oxygenation parameters in patients with ASA physical status 1–2 during general anesthesia undergoing minor elective surgery.

## Background

The EasyTube^®^ (EzT, Well Lead Medical Co., Guangzhou, Panyu, China) is a supraglottic airway device (SAD) that enables effective ventilation irrespective of its placement in the trachea or esophagus [1] and is basically an enhanced version of the Combitube [2, 3]. Its two-lumen design allows ventilation via the proximal lumen and simultaneous placement of a gastric tube via the distal lumen. The EzT has been designed for the “cannot intubate, cannot oxygenate” scenario in prehospital as well as intrahospital settings and is utilized in medical emergency services and emergency departments worldwide [4–6]. It is used not only for anticipated and unanticipated difficult airway situations but also as an airway device used by non-anesthesiologists with limited training in airway management.

Data on supraglottic airway devices are usually obtained from multicenter studies, as those are considered to be the gold standard [7]. Very often in clinical research, conclusions obtained from one highly specialized tertiary care center cannot be extrapolated to other sites or countries, due to local–specific policies, different workflow, education, and experience [8]. A device that provides a benefit at one site, might fail to do so at another. Also, the intention of a principle investigator to investigate a specific device might potently impact the outcome of a study due to personal favoring, availability, and the intention to publish [9–11].

Common practice is to generalize results from such studies whereas detailed data for distinct sites and sites comparison are scarce. The aim of this study was to clarify this topic for the EzT as surrogate for SADs. We compared the use of the EzT in with the ETT at several distinct sites with regards to insertion, ventilation, hemodynamic and ventilatory parameters, and complications. We used endotracheal intubation (ETI) as the gold standard for securing an airway as intra-site control. By using a multicenter database, we aimed to detect site differences with regards to insertion, ventilation, safety, and outcome of intubation with the EzT.

## Methods

The study was approved by the Ethical Committee of the Medical University of Vienna and abided by the Ethical Principles for Medical Research Involving Human Subjects outlined in the Declaration of Helsinki. 400 patients in 4 medical centers (50 patients per center and device; Department of Anesthesiology, Bnai Zion Medical Centre, Haifa, Israel; Department of Anesthesia, Intensive Care Medicine, Emergency Medicine and Pain Therapy, University of Mainz, Germany; Department of Anesthesia and Intensive Care, Mutual de Seguridad Hospital C. CH.C., Santiago, Chile; and Department of Anesthesiology and General Intensive Care, Medical University of Vienna, Vienna, Austria) were included in the study. The data were analyzed anonymously and retrospectively, so no written informed consent was required. In 200 cases, the EzT size 41 French was used. In 200 control patients, conventional ETI was performed using a standard endotracheal tube (ETT) inserted under direct laryngoscopy. All patients had an American Society of Anesthesiologists (ASA) physical status 1 or 2 and were scheduled for extraperitoneal, non-laparoscopic, non-thoracic elective surgery requiring general anesthesia (Table 1). Exclusion criteria included: age < 18 years, acute or chronic lung disease, patients presenting with sore throat, known esophageal disease, oropharyngeal abnormalities, and patients with a cervical spine disease.

**Table 1:** Surgical procedures with the use of EasyTube (EzT) or endotracheal tube (ETT).

	**Site 1**				**Site 2**			
	**EzT**		**ETT**		**EzT**		**ETT**	
	**N**	**%**	**N**	**%**	**N**	**%**	**N**	**%**
**Ankle surgery**	1	2,0	1	2,0	8	16,0	0	0,0
**Appendectomy**	12	24,0	18	36,0	4	8,0	7	14,0
**Arthroscopy**	5	10,0	1	2,0	6	12,0	0	0,0
**Bone biopsy**	0	0,0	0	0,0	0	0,0	0	0,0
**Brest surgery**	8	16,0	3	6,0	3	6,0	4	8,0
**Cystoscopy**	1	2,0	0	0,0	1	2,0	0	0,0
**Hand surgery**	2	4,0	1	2,0	13	26,0	1	2,0
**Hernia**	6	12,0	18	36,0	4	8,0	15	30,0
**Hysterectomy**	0	0,0	6	12,0	0	0,0	2	4,0
**Hysteroscopy**	3	6,0	0	0,0	0	0,0	0	0,0
**Orchiectomy**	1	2,0	1	2,0	0	0,0	0	0,0
**Stent insertion**	2	4,0	0	0,0	0	0,0	3	6,0
**TURP**	2	4,0	0	0,0	8	16,0	2	4,0
**Uretheroscopy**	2	4,0	1	2,0	2	4,0	0	0,0
**Urogenital surgery**	3	6,0	0	0,0	1	2,0	13	26,0
**Varicocelectomy**	2	4,0	0	0,0	0	0,0	3	6,0
	**Site 3**				**Site 4**			
	**EzT**		**ETT**		**EzT**		**ETT**	
	**N**	**%**	**N**	**%**	**N**	**%**	**N**	**%**
**Ankle surgery**	3	6,0	2	4,0	12	24,0	4	8,0
**Appendectomy**	7	14,0	10	20,0	2	4,0	10	20,0
**Arthroscopy**	18	36,0	2	4,0	0	0,0	0	0,0
**Bone biopsy**	2	4,0	0	0,0	0	0,0	0	0,0
**Brest surgery**	0	0,0	3	6,0	1	2,0	5	10,0
**Cystoscopy**	2	4,0	0	0,0	2	4,0	2	4,0
**Hand surgery**	13	26,0	11	22,0	10	20,0	6	12,0
**Hernia**	2	4,0	6	12,0	0	0,0	7	14,0
**Hysterectomy**	0	0,0	2	4,0	0	0,0	2	4,0
**Hysteroscopy**	0	0,0	0	0,0	2	4,0	1	2,0
**Orchiectomy**	0	0,0	0	0,0	0	0,0	0	0,0
**Stent insertion**	0	0,0	1	2,0	2	4,0	5	10,0
**TURP**	2	4,0	1	2,0	8	16,0	3	6,0
**Uretheroscopy**	0	0,0	0	0,0	1	2,0	2	4,0
**Urogenital surgery**	1	2,0	12	24,0	4	8,0	2	4,0
**Varicocelectomy**	0	0,0	0	0,0	6	12,0	1	2,0

Preoperatively, the patients’ airways were classified according to the Mallampati test, patients with suspected difficult airways were excluded [12]. Routine non-invasive monitoring was used including electrocardiogram, pulse oxymetry, non-invasive blood pressure measurement, and capnography. Patients were premedicated with oral midazolam and pre-oxygenated with 100% oxygen. General anesthesia was then induced by an i.v. bolus injection of propofol (1–3 mg/kg) and fentanyl (2–3 μg/kg). Muscle relaxation was achieved using vecuronium bromide. The patient's mouth was opened with one hand and the EzT was inserted in parallel to the patient's chest, so as to facilitate insertion, until the black ring on the EzT was positioned between the patient's maxillary teeth. The EzT was inserted blindly and thereby expected to enter the esophagus with a percentage of about 97%. The oropharyngeal and tracheoesophageal cuffs of the EzT were inflated successively with 80 ml and 5–10 ml, respectively, as recommended by the manufacturer. The ETT was inserted with the help of a Macintosh laryngoscope and the cuff inflated to prevent leakage. Correct placement of the devices was confirmed by auscultation, pulse oxymetry, as well as end-tidal CO_2_-concentration (EtCO_2_) measurements. Simultaneously, insertion difficulty as graded by the anesthesiologist (1–5; 1: very easy, 5: impossible) was documented. Insertion time was measured from grasping the device until confirmation of correct insertion of the EzT or ETT.

After insertion of the EzT or the ETT, anesthesia and ventilatory settings were maintained as required to achieve target oxygenation values of >95%. Patients’ lungs were ventilated with controlled-mode, volume-targeted ventilation. Ventilatory data collected included peak pressure, plateau pressure, PEEP, and inspiratory and expiratory minute volume. Significant leakage was defined as a difference of the inspiratory and the expiratory minute volume of >0.5l/min for more than one minute. When responsive, the patient was questioned for sore throat or dysphagia during postoperative rounds 24 hours after general anesthesia by the physician in charge. The patient received analgesics if required for postoperative pain management on request; this was documented and considered in the documentation of sore throat and dysphagia. Data collection was truncated after one hour for the statistical analysis, as there was significant attrition of data due to the short duration of the procedures.

### Statistical analysis

The primary outcome measure was success of insertion. Secondary outcomes were ease of insertion, ventilation, leakage, hemodynamic data, oxygenation, rate of complications, duration of ventilation, and rating of insertion difficulty by anesthesiologists.

Based on previous studies [13–15], we calculated the necessary sample size with at least 37 participants using G*Power 3.1 (two-tailed t-test; Cohen’s d: 0.8, alpha error: 0.05, power: 0.95). Data were tested for normal distribution and analysis was performed by Chi-squared statistics and unpaired t-tests, respectively. Insertion difficulty as graded by the anesthesiologist, ASA physical status, and Mallampati class were compared using the Kruskal-Wallis test. Bonferroni correction for multiple testing was used. For the primary end point, we used the two one-sided test (TOST) procedure [16]. Results were presented as mean and standard deviation or median and range; a p < 0.05 was considered statistically significant. Log-rank test was used for curve comparison. In case of missing data and loss to follow-up, only the available data were analyzed. Analysis was done with GraphPad Prism 5 (GraphPad Software Inc., CA, USA).

## Results

A total of 200 patients were included into the EzT group, 82 (41%) female. 200 endotracheal intubated patients served as controls (ETT group), 98 (49%) female. Detailed demographic data for each site are depicted in S1 Table, data on surgical procedures are presented in Table 1.

### Insertion

Detailed results for each site are depicted in Table 2. Duration of ventilation did not differ significantly between the devices or between the sites. Overall, the EzT could be inserted within 3 attempts, with a success rate of 82.0% at the first attempt, 17.5% at the second attempt, and 0.5% at the third attempt. The ETT could also be inserted within 3 attempts, with an overall success rate of 88.0% at the first attempt, 11.0% at the second attempt, and 1.0% at the third attempt. Intra-site and inter-site comparison of success rates did not differ significantly (Tables 2 and 3). In all cases, the EzT was placed into the esophagus. Ratings of insertion difficulty of the respective devices at the respective sites are given in Table 2. Centre 2 rated insertion difficulty with the EzT significantly higher (more difficult) than insertion with the ETT, and as compared to the other sites.

**Table 2:** Insertion time, duration of ventilation, number of insertion attempts, and insertion difficulty with the EasyTube (EzT) or endotracheal tube (ETT) in comparison within the sites and in between the sites.

	**Site 1**					**Site 2**				
	**EzT (n = 50)**		**ETT (n = 50)**			**EzT (n = 50)**		**ETT (n = 50)**		
	**Mean**	**SD**	**Mean**	**SD**	**p**	**Mean**	**SD**	**Mean**	**SD**	**p**
**Duration of ventilation (min)**	28,10	13,89	25,19	6,87	n.s.	26,20	15,61	27,20	10,70	n.s.
**Insertion time (sec)**	22,00	4,58	n/a	n/a		22,92	5,29	n/a	n/a	
** **	**N**	**%**	**N**	**%**	**p**	**N**	**%**	**N**	**%**	**p**
**Nr. of insertion attempts**										
**1**	37,00	74,00	48,00	96,00	n.s.	42,00	84,00	46,00	92,00	n.s.
**2**	13,00	26,00	2,00	4,00	n.s.	7,00	14,00	3,00	6,00	n.s.
**3**	0,00	0,00	0,00	0,00	n.s.	0,00	0,00	1,00	2,00	n.s.
**Insertion difficulty**										
**1**	40,00	80,00	46,00	92,00	n.s.	34,00	68,00	40,00	80,00	0.04*
**2**	9,00	18,00	4,00	8,00	n.s.	16,00	32,00	9,00	18,00	0.01*
**3**	0,00	0,00	0,00	0,00	n.s.	0,00	0,00	1,00	2,00	n.s.
	**Site 3**					**Site 4**				
	**EzT (n = 50)**		**ETT (n = 50)**			**EzT (n = 50)**		**ETT (n = 50)**		
	**Mean**	**SD**	**Mean**	**SD**	**p**	**Mean**	**SD**	**Mean**	**SD**	**p**
**Duration of ventilation (min)**	22,20	11,32	24,29	7,28	n.s.	25,20	14,70	23,80	13,46	n.s.
**Insertion time (sec)**	22,36	4,29	n/a	n/a		22,16	3,52	n/a	n/a	
** **	**N**	**%**	**N**	**%**	**p**	**N**	**%**	**N**	**%**	**p**
**Nr. of insertion attempts**										
**1**	43,00	86,00	46,00	92,00	n.s.	41,00	82,00	46,00	92,00	n.s.
**2**	6,00	12,00	4,00	8,00	n.s.	9,00	18,00	3,00	6,00	n.s.
**3**	1,00	2,00	0,00	0,00	n.s.	0,00	0,00	1,00	2,00	n.s.
**Insertion difficulty**										
**1**	41,00	82,00	46,00	92,00	n.s.	43,00	86,00	39,00	78,00	n.s.
**2**	8,00	16,00	4,00	8,00	n.s.	5,00	10,00	9,00	18,00	n.s.
**3**	1,00	2,00	0,00	0,00	n.s.	0,00	0,00	2,00	4,00	n.s.

* values have been corrected for multiple testing

**Table 3:** Insertion time, duration of ventilation, number of insertion attempts, and insertion difficulty with the EasyTube (EzT) or endotracheal tube (ETT) in comparison between the sites.

	Site 1		Site 2		Site 3		Site 4	
	EzT	ETT	EzT	ETT	EzT	ETT	EzT	ETT
**Duration of ventilation (min)**	n.s.	n.s.	n.s.	n.s.	n.s.	n.s.	n.s.	n.s.
**Insertion time (sec)**	n.s.	n.s.	n.s.	n.s.	n.s.	n.s.	n.s.	n.s.
** **								
**Nr. of insertion attempts**								
**1**	n.s.	n.s.	n.s.	n.s.	n.s.	n.s.	n.s.	n.s.
**2**	n.s.	n.s.	n.s.	n.s.	n.s.	n.s.	n.s.	n.s.
**3**	n.s.	n.s.	n.s.	n.s.	n.s.	n.s.	n.s.	n.s.
**Insertion difficulty**								
**1**	n.s.	n.s.	0.04*	n.s.	n.s.	n.s.	n.s.	n.s.
**2**	n.s.	n.s.	0.02*	n.s.	n.s.	n.s.	n.s.	n.s.
**3**	n.s.	n.s.	n.s.	n.s.	n.s.	n.s.	n.s.	n.s.

* values have been corrected for multiple testing

### Ventilation

**Fig 1:**
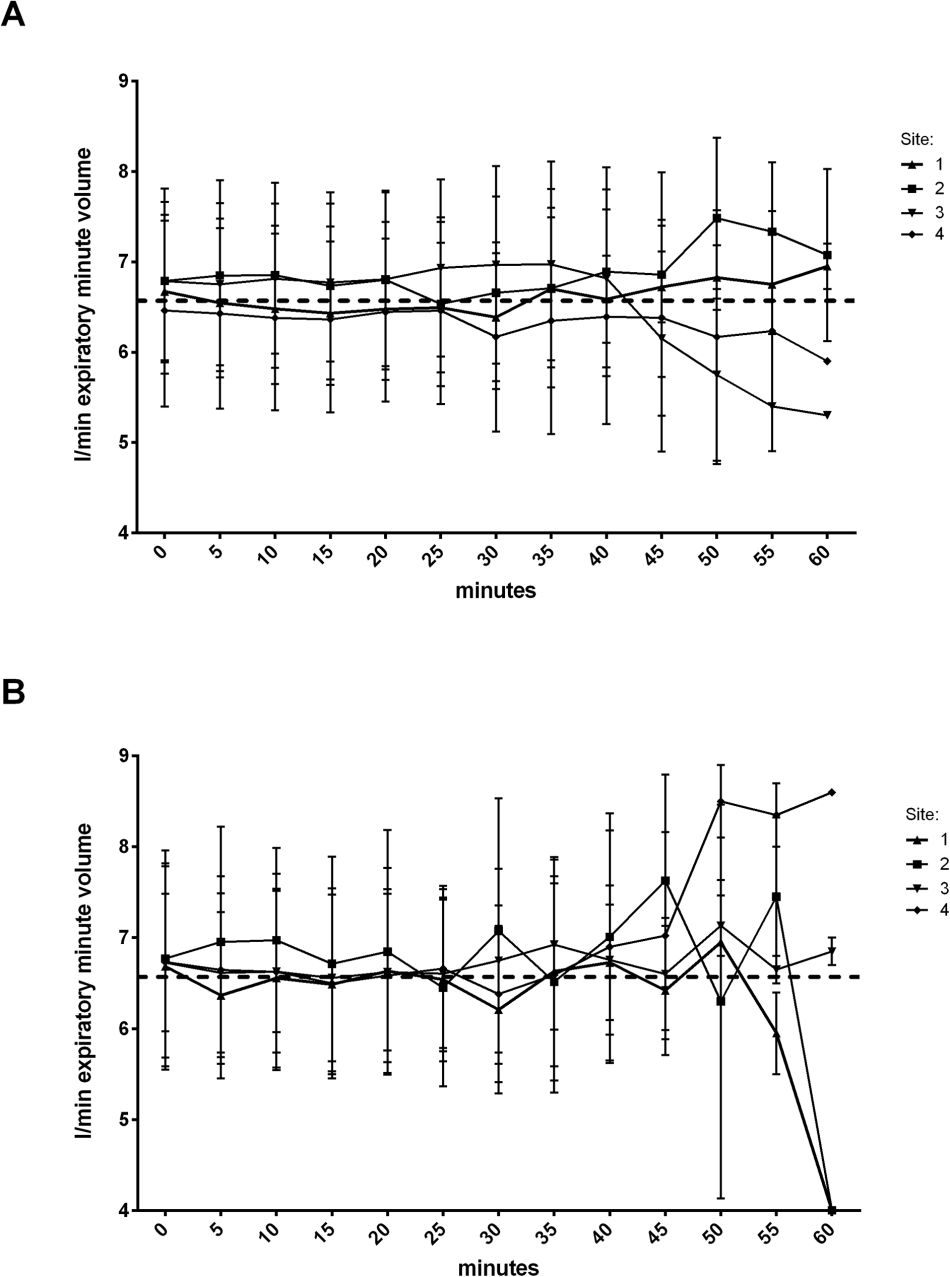
**Expiratory minute volume during general anesthesia with the EzT (A) and the ETT (B) at distinct sites.** Dashed line represents overall mean.

**Fig 2:**
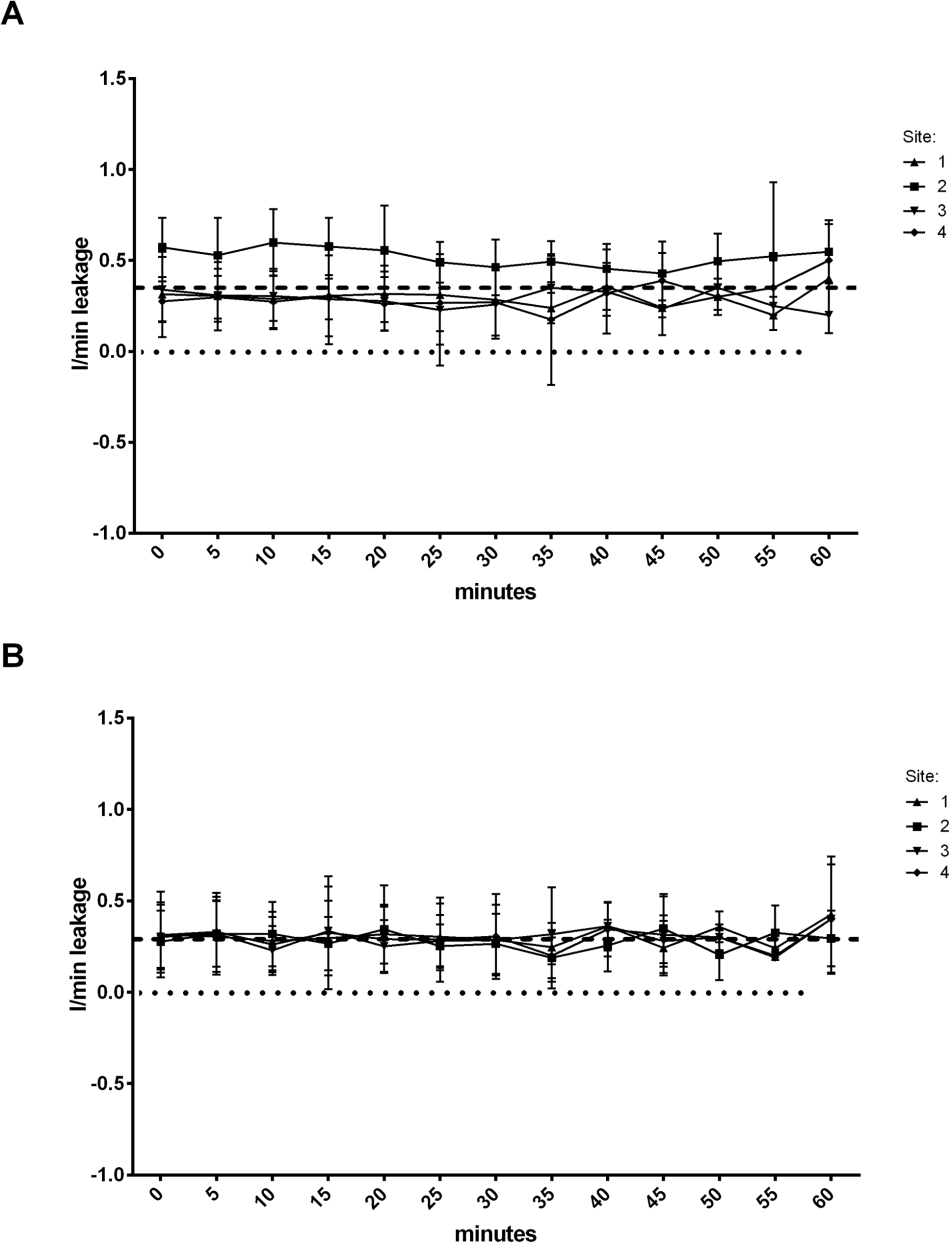
Ventilation leakage of the EzT (A) and the ETT (B) during general anesthesia. Dashed line represents overall mean. Dotted line represents zero.

**Fig 3:**
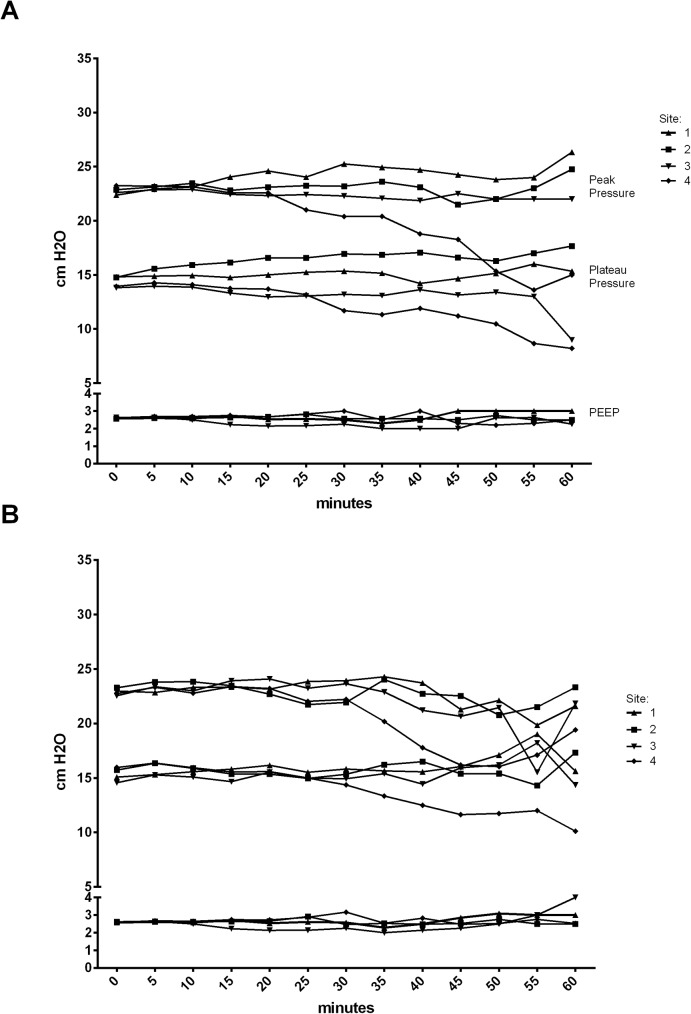
Peak pressure, plateau pressure, and PEEP during general anesthesia with the EzT (A) and the ETT (B).

In all EzT patients the achieved expiratory minute volumes were sufficient to reach target oxygenation values, similar to ETT patients (Fig 1). No significantly different leakage was detected neither in the EzT group nor in the ETT group. However, center 2 reported a significantly higher leakage with the EzT (Fig 2). Mean peak pressure and mean plateau pressure as well as PEEP did not differ between the groups (Fig 3). The inter-site comparison of expiratory minute volumes showed that the volumes achieved over the course of anesthesia did not differ significantly.

### Hemodynamics and oxygenation

No significant differences in heart rate, systolic, and diastolic blood pressure were observed neither in the EzT group nor in the ETT group; there were also no significant variations of heart rate and blood pressure in the course of anesthesia between the sites. Mean SpO_2_ values in the EzT group were 97.98% (± 1.3) and 98.07% (± 1.6) in the ETT patients (p = 0.67). Mean EtCO_2_ values in the EzT group were 37.35 (± 3) mmHg compared to 39.02 (± 3.8) mmHg in the ETT patients. Both SpO_2_ and EtCO_2_ did not show significant differences between the sites.

### Complications

Overall, the most frequent complication was sore throat, followed by the presence of blood on the EzT after removal, and dysphagia (Table 4). The complications were comparable between the devices and centers. However, center 2 showed a significantly higher incidence of blood on balloon after removal (p = 0.01). This correlated in a multivariate analysis with the above finding that at this site, insertion was rated significantly more difficult compared to the on-site control (ETT) and to the other sites. No severe laceration or perforation of the oral, pharyngeal, or esophageal mucosa was detected. In the ETT group, sore throat and blood detected on the cuff on the cuff were the most frequent complications, too, but were recorded to a lesser extent than in the EzT group (Tables 4 and 5).

**Table 4:** Complications during general anesthesia with the EasyTube (EzT) or endotracheal tube (ETT) in comparison within the sites and in between the sites.

	**Site 1**					**Site 2**				
	**EzT (n = 50)**		**ETT (n = 50)**			**EzT (n = 50)**		**ETT (n = 50)**		
** **	**N**	**%**	**N**	**%**	**p**	**N**	**%**	**N**	**%**	**p**
**Sore throat**	12,00	24,00	7,00	14,00	n.s.	12,00	24,00	8,00	16,00	n.s.
**Blood on balloon**	5,00	10,00	5,00	10,00	n.s.	19,00	38,00	6,00	12,00	n.s.
**Dysphagia**	0,00	0,00	0,00	0,00	n.s.	2,00	4,00	2,00	4,00	n.s.
**Tongue edema**	0,00	0,00	0,00	0,00	n.s.	1,00	2,00	1,00	2,00	n.s.
**Mucosal lesions**	0,00	0,00	1,00	2,00	n.s.	1,00	2,00	0,00	0,00	n.s.
**Tooth breakage**	0,00	0,00	0,00	0,00	n.s.	0,00	0,00	0,00	0,00	n.s.
**Occlusion of the pharyngeal lumen**	0,00	0,00	n/a	n/a		0,00	0,00	n/a	n/a	
	**Site 3**					**Site 4**				
	**EzT (n = 50)**		**ETT (n = 50)**			**EzT (n = 50)**		**ETT (n = 50)**		
** **	**N**	**%**	**N**	**%**	**p**	**N**	**%**	**N**	**%**	**p**
**Sore throat**	10,00	20,00	10,00	20,00	n.s.	11,00	22,00	7,00	14,00	n.s.
**Blood on balloon**	10,00	20,00	12,00	24,00	n.s.	10,00	20,00	6,00	12,00	n.s.
**Dysphagia**	3,00	6,00	3,00	6,00	n.s.	0,00	0,00	0,00	0,00	n.s.
**Tongue edema**	1,00	2,00	0,00	0,00	n.s.	2,00	4,00	0,00	0,00	n.s.
**Mucosal lesions**	2,00	4,00	2,00	4,00	n.s.	0,00	0,00	2,00	4,00	n.s.
**Tooth breakage**	0,00	0,00	1,00	2,00	n.s.	0,00	0,00	0,00	0,00	n.s.
**Occlusion of the pharyngeal lumen**	0,00	0,00	n/a	n/a		0,00	0,00	n/a	n/a	

**Table 5:** Complications during general anesthesia with the EasyTube (EzT) or endotracheal tube (ETT) in comparison between the sites.

	Site 1		Site 2		Site 3		Site 4	
	EzT	ETT	EzT	ETT	EzT	ETT	EzT	ETT
**Sore throat**	n.s.	n.s.	n.s.	n.s.	n.s.	n.s.	n.s.	n.s.
**Blood on balloon**	n.s.	n.s.	0.01*	n.s.	n.s.	n.s.	n.s.	n.s.
**Dysphagia**	n.s.	n.s.	n.s.	n.s.	n.s.	n.s.	n.s.	n.s.
**Tongue edema**	n.s.	n.s.	n.s.	n.s.	n.s.	n.s.	n.s.	n.s.
**Mucosal lesions**	n.s.	n.s.	n.s.	n.s.	n.s.	n.s.	n.s.	n.s.
**Tooth breakage**	n.s.	n.s.	n.s.	n.s.	n.s.	n.s.	n.s.	n.s.
**Occlusion of the pharyngeal lumen**	n.s.	n.s.	n.s.	n.s.	n.s.	n.s.	n.s.	n.s.

* values have been corrected for multiple testing

## Discussion

To our knowledge, this is the first study focusing on inter-site variability of a SAD in a multicenter setting. The most important finding of our study is that insertion, ventilation, hemodynamics, and oxygenation of the EzT were comparable to the ETT and did not differ significantly between the sites. This is in accordance with overall results from other studies [14, 15, 17, 18]. We further show for the first time that there is a significant difference in rate of complications, leakage, and in difficulty of insertion amongst the different sites. This is important when interpreting results from multi-center studies, drafting standard-operating-procedures, and for airway management training as it shows that conclusions obtained from one highly specialized tertiary care center cannot be directly transferred to other sites without adaption to the distinct situation on site.

The insertion success rates for both devices were comparable, with a slightly higher success rate with the ETT. Also, there was no difference between the sites. This might possibly be due to the fact that anesthesiologists participating in the study were more experienced with ETI with an ETT. Also, training in airway management is similar though not standardized worldwide. The observed insertion success rates for the EzT and ETT were comparable to other studies (Sethi 87% [14], Gaitini 92.5% [18], Lorenz 95% [15], Cavus 64% [13], Park [19]).

The expiratory minute volumes in our study were comparable to the results obtained previously by Gaitini [17] and did not differ between the groups. However, we detected a mean gas leakage of 0.29 L/min whereas Gaitini et al [12] reported a mean leakage of 0.38 L/min. Importantly, leakage at site 2 was significantly higher than at the other sites. However, the available data cannot provide a reason for this. We can therefore only speculate that maybe the oropharyngeal cuff was not inflated properly which might be caused by either lack of experience with the device or lack of initial training. Also, different ventilator types might contribute to the observed difference. In addition, as shown by Park et al the head and neck position impacts the oropharyngeal leak pressure, cuff position, and leakage [20].

The similarly designed Combitube has significant airflow resistance that should be considered when patients are mechanically ventilated [21]. We therefore expected higher ventilatory pressures in the EzT group. However, peak and mean airway pressures were in accordance to previously reported data [14, 18], but lower than reported for the Combitube [21]. Also, we did not observe a significant change in peak or mean airway pressure compared to baseline as reported by Sethi [14]. Furthermore, we did not observe changes in hemodynamic parameters in our patients during the course of anesthesia as previously reported [14]. It is also questionable whether the changes observed by Sethi can be attributed to a specific airway device or rather to insufficient depth of anesthesia or insertion technique. Oxygenation was appropriate at all sites with no significant difference between the sites.

The overall incidence of dental injury in the literature is reported between 0.06% and 12% with the ETT, which is in accordance with our data [22–26]. The risk of dental injury is practically non-existing with EzT insertion, as it is usually inserted without utilizing a laryngoscope. Other complications were minor and not significantly different between the sites except for blood on balloon. While this specific complication was more frequent in the EzT group at site 2, a broken tooth might cause more severe problems. Of note, sore throat also was more frequent at site 2, but without reaching statistical significance. This might be explained by the placement of the distal balloon in the esophagus and by the relatively large pharyngeal balloon sitting in the oropharynx which might cause discomfort [27]. Anesthesiologists usually get better with handling intubation devices with each try. Therefore, the learning curve at site 2 could be useful in explaining the difficulty in insertion of the EzT as experience might impact the rate of complication and the insertion success. Since we do not have detailed data on the level of experience, medical education, and experience with the respective device of the anesthesiologists, any conclusion would be speculation.

A limitation of our study is that we included only patients with an ASA physical status 1–2, which hinders generalization of the results to critically ill patients. Also, the participants underwent minor surgical procedures only (Table 1). Furthermore, the investigated time period was one hour, so no conclusions for longer-lasting ventilation can be drawn from our data. Another limitation of this study is that only patients with anticipated normal airways were included, and thus the performance and rate of complications of the EzT used as a rescue device in emergency situations remains unclear. Also, due to the retrospective design, there is a possibility that some information is missing or reported falsely in the database, which might contribute to the variability in some centers. There is currently only one randomized, clinical study on intubation with the EzT that reported 14 patients with a difficult airway [4], and one manikin study of our own group addressing this important topic [28]. Therefore, further clinical research on the use of the EzT in difficult airway situations is warranted [29].

In conclusion, performance of the EzT but not the ETT varied between sites with regard to insertion difficulty, leakage, and complications but not insertion success, ventilation, hemodynamics, and oxygenation parameters in patients with normal airways during general anesthesia undergoing minor elective surgery. When interpreting results from multicenter studies on SAD’s, site-specific practices should be considered.

## Supporting information

S1 Table Demographic data of patients with use of EasyTube (EzT) or endotracheal tube (ETT) at each site. (DOCX)
