# Supplementary material for: Inter-center comparison of EasyTube and endotracheal tube during general anesthesia in minor elective surgery
Source: PLoS One. 2017 Jun 2;12(6):e0178756. doi: 10.1371/journal.pone.0178756 (PMC5456362; doi:10.1371/journal.pone.0178756)
Supplement: S1 Table — (DOCX) [file pone.0178756.s001.docx]

**S1 Table: Demographic data of patients with use of EasyTube (EzT) or endotracheal tube (ETT) at each site**

|  | **Site 1** | | | | | **Site 2** | | | | |
| --- | --- | --- | --- | --- | --- | --- | --- | --- | --- | --- |
|  | **EzT (n=50)** | | **ETT (n=50)** | |  | **EzT (n=50)** | | **ETT (n=50)** | |  |
|  | **Mean** | **SD** | **Mean** | **SD** | **p** | **Mean** | **SD** | **Mean** | **SD** | **p** |
| **Age (years)** | 55,56 | 16,65 | 66,88 | 16,59 | n.s. | 52,30 | 16,72 | 57,56 | 21,00 | n.s. |
| **Height (cm)** | 172,66 | 6,70 | 168,20 | 8,45 | n.s. | 172,70 | 7,48 | 172,28 | 11,28 | n.s. |
| **Weight (kg)** | 74,90 | 9,77 | 79,81 | 21,60 | n.s. | 70,80 | 11,37 | 77,96 | 20,80 | n.s. |
| **BMI (kg/m²)** | 25,08 | 2,64 | 28,60 | 7,83 | n.s. | 23,46 | 3,35 | 26,58 | 6,18 | n.s. |
|  | **N** | **%** | **N** | **%** |  | **N** | **%** | **N** | **%** |  |
| **Female** | 16 | 32 | 30 | 60 | n.s. | 17 | 34 | 21 | 41 | n.s. |
| **Male** | 34 | 68 | 20 | 40 | n.s. | 33 | 66 | 30 | 59 | n.s. |
| **ASA** |  |  |  |  |  |  |  |  |  |  |
| **1** | 33 | 66 | 10 | 20 | n.s. | 35 | 70 | 19 | 38 | n.s. |
| **2** | 17 | 34 | 40 | 80 | n.s. | 15 | 30 | 31 | 62 | n.s. |
| **Mallampati** |  |  |  |  |  |  |  |  |  |  |
| **1** | 36 | 72 | 33 | 66 | n.s. | 42 | 84 | 39 | 78 | n.s. |
| **2** | 14 | 28 | 10 | 20 | n.s. | 8 | 16 | 7 | 14 | n.s. |
| **3** | 0 | 0 | 0 | 0 | n.s. | 0 | 0 | 0 | 0 | n.s. |
|  | **Site 3** | | | | | **Site 4** | | | | |
|  | **EzT (n=50)** | | **ETT (n=50)** | |  | **EzT (n=50)** | | **ETT (n=50)** | |  |
|  | **Mean** | **SD** | **Mean** | **SD** | **p** | **Mean** | **SD** | **Mean** | **SD** | **p** |
| **Age (years)** | 52,42 | 17,47 | 59,50 | 20,91 | n.s. | 54,94 | 14,83 | 65,72 | 16,55 | n.s. |
| **Height (cm)** | 172,31 | 7,98 | 172,34 | 8,14 | n.s. | 171,06 | 7,02 | 167,32 | 8,25 | n.s. |
| **Weight (kg)** | 73,44 | 10,58 | 75,72 | 23,07 | n.s. | 68,28 | 9,37 | 78,97 | 20,73 | n.s. |
| **BMI (kg/m²)** | 24,65 | 3,25 | 25,75 | 6,49 | n.s. | 23,30 | 2,70 | 28,92 | 8,18 | n.s. |
|  | **N** | **%** | **N** | **%** |  | **N** | **%** | **N** | **%** |  |
| **Female** | 21 | 42 | 22 | 43 | n.s. | 28 | 56 | 31 | 62 | n.s. |
| **Male** | 29 | 58 | 29 | 57 | n.s. | 22 | 44 | 19 | 38 | n.s. |
| **ASA** |  |  |  |  |  |  |  |  |  |  |
| **1** | 31 | 62 | 13 | 26 | n.s. | 33 | 66 | 12 | 24 | n.s. |
| **2** | 19 | 38 | 37 | 74 | n.s. | 17 | 34 | 38 | 76 | n.s. |
| **Mallampati** |  |  |  |  |  |  |  |  |  |  |
| **1** | 39 | 78 | 36 | 72 | n.s. | 44 | 88 | 38 | 76 | n.s. |
| **2** | 11 | 22 | 9 | 18 | n.s. | 5 | 10 | 8 | 16 | n.s. |
| **3** | 0 | 0 |  | 0 | n.s. | 1 | 2 | 4 | 8 | n.s. |
